# Association of *PIK3CA* Mutation With Pathologic Complete Response and Outcome by Hormone Receptor Status and Intrinsic Subtype in Early-Stage ERBB2/HER2-Positive Breast Cancer

**DOI:** 10.1001/jamanetworkopen.2023.48814

**Published:** 2023-12-20

**Authors:** Paola Zagami, Aranzazu Fernandez-Martinez, Naim U. Rashid, Katherine A. Hoadley, Patricia A. Spears, Giuseppe Curigliano, Charles M. Perou, Lisa A. Carey

**Affiliations:** 1 Lineberger Comprehensive Cancer Center, University of North Carolina, Chapel Hill; 2 Division of Medical Oncology, University of Milan, Milan, Italy; 3 Department of Genetics, University of North Carolina, Chapel Hill; 4 Department of Biostatistics, University of North Carolina, Chapel Hill; 5 Division of New Drugs and Early Drug Development, European Institute of Oncology IRCCS, Milan, Italy; 6 Division of Medical Oncology, Department of Medicine, School of Medicine, University of North Carolina at Chapel Hill, Chapel Hill

## Abstract

**Importance:**

*PIK3CA* mutations may be associated with outcomes of patients with ERBB2/HER2-positive early breast cancer (EBC).

**Objectives:**

To assess if *PIK3CA* mutations among patients with ERBB2/HER2-positive EBC are associated with treatment response and outcome, and if these associations vary by hormone receptor (HR) status or intrinsic molecular subtype (IMS).

**Design, Setting, and Participants:**

This cohort study derived data on 184 patients from the phase 3 neoadjuvant Cancer and Leukemia Group B (CALGB) 40601 trial that enrolled patients with ERBB2/HER2-positive EBC in North America between January 1, 2008, and December 31, 2012. Participants received neoadjuvant paclitaxel with trastuzumab, lapatinib, or both. Statistical analysis was performed from March 23, 2022, to March 9, 2023.

**Exposures:**

Gene expression profiling by RNA sequencing with Prediction Analysis of Microarray 50–determined IMS and *PIK3CA* mutations from whole-exome sequencing were obtained from pretreatment biopsies from 184 of 305 trial participants.

**Main Outcomes and Measures:**

The primary end point was pathologic complete response (pCR) and the secondary end point of event-free survival (EFS). The association of *PIK3CA* mutations with pCR and EFS by HR status and IMS was estimated using logistic and Cox proportional hazards regression models.

**Results:**

All 184 participants were women, with a median age of 49 years (range 24-75 years). A total of 121 participants (66%) had clinical stage II tumors; 32 (17%) had *PIK3CA* mutations, most frequently H1047R (38% [12 of 32]) and E545K (22% [7 of 32]). *PIK3CA* mutations were present in 20 of 102 cases of HR-positive EBC (20%) and 12 of 82 cases HR-negative EBC (15%) and varied by IMS (luminal B, 9 of 25 [36%]; luminal A, 2 of 21 [10%]; and ERBB2/HER2-enriched tumors, 19 of 102 [19%]). Pathologic complete response rates were lower in *PIK3CA* mutated than *PIK3CA* wild type in the overall population (34% [11 of 32] vs 49% [74 of 152]; *P* = .14) and were significantly different among those receiving trastuzumab (30% [7 of 23] vs 54% [63 of 117]; *P* = .045). At a median follow-up of 9 years, *PIK3CA* mutations were significantly associated with worse EFS in the overall cohort (hazard ratio, 2.58 [95% CI, 1.24-5.35]; *P* = .01), which persisted in a multivariable model including pCR, HR status, stage, and IMS (hazard ratio, 2.52 [95% CI, 1.16-5.47]; *P* = .02). The negative association of *PIK3CA* mutation was significant in HR-positive (hazard ratio, 3.60 [95% CI, 1.45-8.96]; *P* = .006) and luminal subtypes (hazard ratio, 4.84 [95% CI, 1.08-21.70]; *P* = .04), but not in nonluminal and HR-negative tumors.

**Conclusions and Relevance:**

In ERBB2/HER2-positive EBC, *PIK3CA* mutations were associated with lower pCR rates and independently associated with worse long-term EFS. These findings appear to be associated with *PIK3CA* mutations in HR-positive and luminal EBC.

## Key Points

**Question:**

What is the association of *PIK3CA* mutations, response to therapy, and outcome by hormone receptor (HR) status and intrinsic subtype among patients with ERBB2/HER2-positive early breast cancer (EBC) treated in a clinical trial?

**Findings:**

In this cohort study of 184 patients enrolled in the phase 3 trial CALGB 40601, *PIK3CA* mutations were associated with lower pathologic complete response rates to chemotherapy plus trastuzumab and independently worse event-free survival, owing to the HR-positive and luminal intrinsic subtypes.

**Meaning:**

This study suggests that *PIK3CA* mutations, which are therapeutically targetable, are associated with response to therapy as well as outcome in ERBB2/HER2-positive EBC, particularly luminal disease.

## Teaser

This cohort study assesses whether *PIK3CA* mutations among patients with ERBB2/HER2-positive early breast cancer are associated with treatment response and outcome, and if these associations vary by hormone receptor status or intrinsic molecular subtype.

## Introduction

ERBB2/HER2 overexpression or amplification defines the clinical ERBB2/HER2-positive breast cancer (BC) subtype. The introduction in clinical practice of ERBB2/HER2-targeted therapies has changed the natural history of ERBB2/HER2-positive BC. A long history of clinical (neo)adjuvant studies investigated the use of dual ERBB2/HER2 blockade to increase pathologic complete response (pCR) rates and improve outcomes in ERBB2/HER2-positive early BC (EBC). Currently, neoadjuvant chemotherapy plus dual blockade (trastuzumab plus pertuzumab, for a total of 1 year) represents the standard of care for ERBB2/HER2-positive EBC; however, response to treatment and survival outcomes remain highly variable.

In 30% to 35% of ERBB2/HER2-positive BCs, the protein phosphatidylinositol 3-kinase (PI3K) pathway, involved in ERBB2/HER2 oncogene signaling,^1^ may be altered, mostly in the *PIK3CA* gene (OMIM 164870), encoding the PI3K α-catalytic subunit.^2^ In ERBB2/HER2-positive BC, different hotspot *PIK3CA* mutations (most frequently in exons 9 and 20^2^) have been associated with ERBB2/HER2-independent activation of the PI3K pathway and resistance to anti-ERBB2/HER2 agents.^1,3^ As new anti-ERBB2/HER2 drugs were developed, new predictive and prognostic biomarkers have been developed, including intrinsic molecular subtype (IMS), immune gene expression,^4^ and tumor-infiltrating lymphocyte counts,^5^ as the most promising to tailor treatment. *PIK3CA* mutations were associated with reduced pCR rates after neoadjuvant anti-ERBB2/HER2–based therapy, with mixed data regarding long-term outcomes.^3,6,7,8,9^ The implication of *PIK3CA* mutations in treatment response heterogeneity, long-term outcomes, and interaction with IMS within ERBB2/HER2-positive EBC is still unknown. Here, we studied the prognostic implications of *PIK3CA* mutations by hormone receptor (HR) status and IMS in the neoadjuvant Cancer and Leukemia Group B (CALGB) 40601 trial. Cancer and Leukemia Group B is now part of the Alliance for Clinical Trials in Oncology.

## Methods

The CALGB 40601 trial design and end points have been previously published.^10,11,12^ This phase 3 trial randomized patients with newly diagnosed, untreated stage II or III ERBB2/HER2-positive EBC from 318 US and Canadian study locations between January 1, 2008, and December 31, 2012, to neoadjuvant weekly paclitaxel with trastuzumab, lapatinib, or both. Anthracycline-based chemotherapy plus completion of 1 year of trastuzumab was recommended after surgery. ERBB2/HER2 testing was locally performed; positivity was defined by the trial eligibility as 3+ by immunohistochemistry (or 2+ with gene amplification by fluorescence in situ hybridization with a ratio of ≥2.0). Hormone receptor status was defined by local laboratory standards following American Society of Clinical Oncology/College of American Pathologists (ASCO/CAP) guidelines.^13^ This analysis followed Reporting Recommendations for Tumor Marker Prognostic Studies (REMARK) guideline criteria^14^ and the Strengthening the Reporting of Observational Studies in Epidemiology (STROBE) reporting guideline. The National Cancer Institute Central Institutional Review Board approved this study. Patients signed a written institutional review board–approved, protocol-specific informed consent including biomarker research following federal and institutional guidelines.

A total of 305 patients were enrolled in CALGB 40601 and underwent 4 pretreatment 16-gauge core biopsies for research purposes, of whom 184 had adequate samples for DNA and RNA sequencing (RNASeq) (eFigure 1 in Supplement 1) and serve as this correlative study cohort. Whole-transcriptome analyses by RNASeq and whole exome sequencing were performed as specified (eMethods in Supplement 1), and all data are at the Database of Genotypes and Phenotypes (phs001570.v3.p1) and Gene Expression Omnibus (GSE116335). For gene expression profiling, whole-transcriptome analyses by RNASeq were performed in the University of North Carolina (UNC) High-Throughput Sequencing Facility and analyzed by the UNC Lineberger Comprehensive Cancer Center Bioinformatics Core.

The ethnic categories in the CALGB 40601 clinical trial protocol were Hispanic or Latino, not Hispanic or Latino, and not available. The racial categories included in the protocol were Black or African American, White, and other (including American Indian or Alaska Native, Asian, Native Hawaiian or Other Pacific Islander, and not available). In our cohort, 8 patients classified as “other” did not have information on race available. The ethnic distribution for these 8 patients were as follows: 6 non-Hispanic, 1 Hispanic, and 1 not available.

### Statistical Analysis

Statistical analysis was performed from March 23, 2022, to March 9, 2023. Tumor and patient characteristics were analyzed using descriptive statistics. Event-free survival (EFS) was calculated from surgery to first recurrence (locoregional or distant) or death from any cause, and the median follow-up was 9.1 years (IQR, 8.0-9.9 years), with the study clinical database frozen on June 10, 2021. Standard of care treatment of ERBB2/HER2-positive EBC includes trastuzumab; the lapatinib-only group was investigational, found inferior in several studies including this one, and was closed early.^15,16^ Response to therapy and prognostic analyses of *PIK3CA* mutation were preplanned in CALGB 40601; these were performed both in the overall cohort and, in an exploratory analysis, only in those treated with trastuzumab-based therapy, excluding those treated in the lapatinib group, as has been done previously.^10^ Logistic and Cox proportional hazards regression analyses were used to associate *PIK3CA* mutations with outcomes.

Hazard ratios, odds ratios (ORs), and 95% CIs were calculated and reported. The Kaplan-Meier method estimated 9-year EFS. All statistical tests were 2-sided, with *P* < .05 as significance level and performed using R, version 4.1.3 (R Project for Statistical Computing), and Python, version 3.6 (Python Software Foundation).

## Results

All 184 trial participants included in this cohort were women and largely White (149 [81%]); 13 (7%) were self-identified Black (Table 1). Median age was 49 years (range, 24-75 years), and 121 (66%) had stage II EBC. Approximately 55% had HR-positive BC. The most frequent intrinsic subtype in both the mutated *PIK3CA* (19 of 32 [59%]) and wild-type (wt) *PIK3CA* (83 of 152 [55%]) groups was ERBB2/HER2 enriched.

**Table 1.** Clinicopathologic Characteristics of Patients by *PIK3CA* Mutation Status

Characteristic	No./total No. (%)			*P* value^a^
	Total cohort (N = 184)	*PIK3CA* wild type (n = 152)	*PIK3CA* mutated (n = 32)	
Age, median (range), y	49 (24-75)	49 (42-56)	51 (42-59)	.50
Menopausal status				
Postmenopausal	78/184 (42)	64/78 (82)	14/78 (18)	.90
Premenopausal	106/184 (58)	88/106 (83)	18/106 (17)	
Race and ethnicity				
Asian	14/184 (8)	12/14 (86)	2/14 (14)	.40
Black	13/184 (7)	9/13 (69)	4/13 (31)	
White	149/184 (81)	125/149 (84)	24/149 (16)	
Other^b^	8/184 (4)	6/8 (75)	2/8 (25)	
Hormone receptor status				
Negative	82/184 (45)	70/82 (85)	12/82 (15)	.40
Positive	102/184 (55)	82/102 (80)	20/102 (20)	
Clinical stage				
II	121/184 (66)	93/121 (77)	28/121 (23)	.004
III	63/184 (34)	59/63 (94)	4/63 (6)	
Subtype				
Basal	15/184 (8)	14/15 (93)	1/15 (7)	.047
ERBB2/HER2 enriched	102/184 (55)	83/102 (81)	19/102 (19)	
Luminal A	21/184 (11)	19/21 (91)	2/21 (10)	
Luminal B	25/184 (14)	16/25 (64)	9/25 (36)	
Normal	21/184 (11)	20/21 (95)	1/21 (5)	

^a^ The distributions or categorical covariates were compared using the Fisher exact test.

^b^ Included American Indian or Alaska Native, Asian, Native Hawaiian or Other Pacific Islander, and not available. In our cohort, the 8 patients classified as “other” did not have information on race available.

*PIK3CA* mutations were detected in 32 patients (17%). The most frequent mutations were H1047R (12 of 32 [38%]), E545K (7 of 32 [22%]), and E542K (5 of 32 [16%]) (eFigure 2 in Supplement 1). The clinicopathologic characteristics of the patients within the 2 groups (ie, *PIK3CA*-mutated and *PIK3CA* wt) are described in Table 1. Within luminal A, luminal B, and ERBB2/HER2-enriched BC, *PIK3CA* mutations occurred among 2 of 21 cases (10%), 9 of 25 cases (36%), and 19 of 102 cases (19%), respectively (eFigure 3 in Supplement 1).

### *PIK3CA* Mutation and pCR

**Figure 1.**
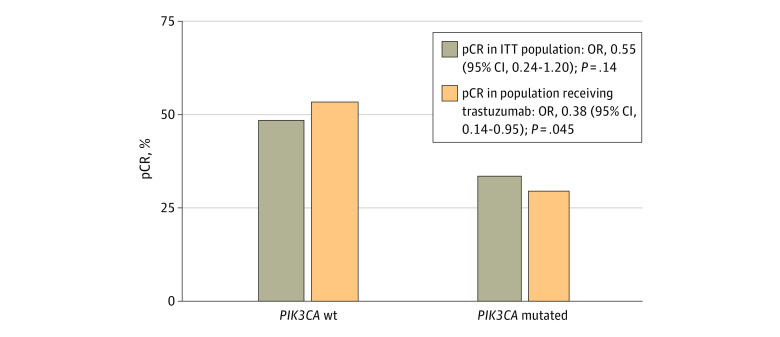
Pathologic Complete Response (pCR) Rate of by *PIK3CA* Mutation in the Intention-to-Treat (ITT) Population and Patients Receiving Trastuzumab-Based Therapy OR indicates odds ratio; wt, wild type.

There was a numerically lower pCR rate in patients with *PIK3CA*-mutated tumors than those with *PIK3CA* wt tumors (34% [11 of 32] vs 49% [74 of 152]; OR, 0.55 [95% CI, 0.24-1.20]; *P* = .14). When limited to patients treated with paclitaxel plus trastuzumab-based therapy (only trastuzumab or trastuzumab plus lapatinib), excluding the lapatinib-only group that was stopped early for futility and toxic effects, the pCR rate among *PIK3CA*-mutated tumors was significantly lower (30% [7 of 23] vs 54% [63 of 117]; OR, 0.38 [95% CI, 0.14-0.95]; *P* = .045) (Figure 1).

### *PIK3CA* Mutation and EFS by HR Status and Intrinsic Subtype

**Figure 2.**
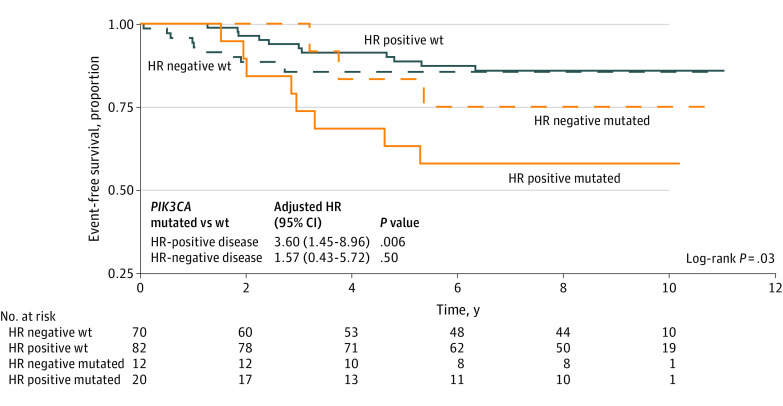
Event-Free Survival by *PIK3CA* Mutation and Hormone Receptor (HR) Status Mutated indicates *PIK3CA* mutated; wt, *PIK3CA* wild type.

At a median follow-up of 9 years, the presence of *PIK3CA* mutation was significantly associated with worse EFS in the study population in a univariable model (hazard ratio, 2.58 [95% CI, 1.24-5.35]; *P* = .01) (eFigure 4 in Supplement 1) and a multivariable model including pCR status, HR status, stage, and IMS (adjusted hazard ratio, 2.52 [95% CI, 1.16-5.47]; *P* = .02) (Table 2). The negative association of *PIK3CA* mutation with EFS appeared stronger among patients with HR-positive (hazard ratio, 3.60 [95% CI, 1.45-8.96]; *P* = .006) than HR-negative disease (hazard ratio, 1.57 [95% CI, 0.43-5.72]; *P* = .50) and appeared to be associated with poor outcome in HR-positive and *PIK3CA*-mutated disease (Figure 2). This finding remained significant in a stratified multivariable model (HR positive: adjusted hazard ratio, 4.84 [95% CI, 1.63-14.30]; *P* = .004; HR negative: hazard ratio, 1.36 [95% CI, 0.36-5.11]; *P* = .68). Analysis of EFS by IMS also found excellent outcome in wt combined luminal A and B tumors, with significantly worse outcome in *PIK3CA*-mutated tumors (hazard ratio, 4.84 [95% CI, 1.08-21.70]; *P* = .04) (eFigure 5 in Supplement 1).

**Table 2.** Multivariable Model for Event-Free Survival

Characteristic	Hazard ratio (95% CI)	*P* value
*PIK3CA*		
Wild type	[Reference]	.02
Mutated	2.52 (1.16-5.47)	
Pathologic response		
pCR	[Reference]	.01
RD	2.94 (1.26-6.87)	
Clinical stage		
II	[Reference]	.14
III	1.77 (0.82-3.80)	
Intrinsic subtype		
Not ERBB2/HER2 enriched	[Reference]	.20
ERBB2/HER2 enriched	1.65 (0.76-3.60)	
Hormone receptor status		
Negative	[Reference]	.70
Positive	1.15 (0.53-2.49)	

Abbreviations: pCR, pathologic complete response; RD, residual disease.

## Discussion

We investigated the association of *PIK3CA* mutations in ERBB2/HER2-positive EBC with pathologic response to therapy and outcome by IMS and HR status in the phase 3 trial CALGB 40601 and found that *PIK3CA* mutations were associated with lower pCR rates among trastuzumab-treated patients and were independently associated with poorer 9-year EFS owing to associations in HR-positive and luminal subtypes. Our finding of worse pCR rates among patients with *PIK3CA*-mutated tumors is concordant with other trials^3,6,17^ and with a pooled analysis of 5 neoadjuvant trials using the same drugs as in CALGB 40601 (trastuzumab with or without lapatinib).^3^

The literature regarding the association of *PIK3CA* mutation in ERBB2/HER2-positive disease with pCR is consistent; however, studies of *PIK3CA* and survival have had mixed results.^3,6,7,8,9^ These studies examining *PIK3CA* mutation and outcome differ from each other, and from our trial, by size, treatment type, length of follow-up, and DNA sequencing methods. In the phase 3 Short-Her adjuvant trial, no association of *PIK3CA* mutation and 5-year disease-free survival (DFS) was seen even when limited to patients receiving the standard 1 year of trastuzumab.^8^ That trial had a different distribution of IMS and *PIK3CA* mutations from CALGB 40601. Similarly, *PIK3CA* mutations in the Finland Herceptin adjuvant trial did not show a prognostic effect^7^; however, the follow-up was only 5 years, and there was a trend toward worse DFS after 3 years. That population also included patients who did not receive adjuvant trastuzumab. In a pooled analysis of 5 randomized trials, DFS was reported at just under 4 years of follow-up, finding no significant difference in outcome by *PIK3CA* mutation status in the overall population.^3^ However, like CALGB 40601, significantly worse DFS was noted among the subgroup with *PIK3CA*-mutated, HR-positive disease. This finding may reflect antiestrogen resistance, which is well documented in the HR-positive or ERBB2/HER2 setting, and its importance for patients with HR-positive disease regardless of ERBB2/HER2, which may require long follow-up.^18^ This analysis of CALGB 40601 has a median follow-up of 9 years, substantially longer than in the other studies. This long follow-up may be more relevant for events in the luminal and HR-positive subsets that appear to be key to the association with outcomes, and in which later recurrences are common.

Most previously published data^6,7,8^ on *PIK3CA* mutations in ERBB2/HER2-positive EBC used DNA sequencing methods (eg, Sanger or pyrosequencing) based on commercial assays that can detect a specific number of well-characterized mutant sequences, which may result in lower sensitivity than whole-exome sequencing used in CALGB 40601. The whole-exome sequencing detection method used in this cohort study was updated to the genome hg38, and somatic variants that were identified were called by 3 different methods (instead of 1, as previously reported^12^), ostensibly augmenting sensitivity; some mutations such as N345K are poorly detected by commercial panels but visible by whole-exome sequencing.^2^ However, the *PIK3CA* mutation rate noted in this study (17%) is not higher than in large pooled neoadjuvant data sets (21.7%) that largely used Sanger sequencing, Sequenom genotyping, or pyrosequencing.^3^

### Limitations

This cohort study has some limitations, including that it represents a retrospective analysis of CALGB 40601 that includes a subset of the overall enrolled patient population. ERBB2/HER2 status and HR expression were not centrally reviewed. The trial tested dual vs single anti-ERBB2/HER2 therapy, with the preoperative component including a single chemotherapy agent, paclitaxel. This trial presaged current efforts to limit chemotherapy, and actually mimics the approach being taken in de-escalation trials such as ECOG/ACRIN EA1181 (NCT04266249). However, CALGB 40601 used lapatinib as the dual anti-ERBB2/HER2 agent, which does not reflect the current standard of care in which the ERBB2/HER2 monoclonal antibody pertuzumab is the dual agent added to trastuzumab. Modern regimens include adjuvant trastuzumab emtansine (T-DM1) for those with residual disease at surgery; this regimen was not used in CALGB 40601’s era and is both a limitation and a strength because prognosis omitting T-DM1, which has considerable toxic effects and expense, is also an unmet research need. The parent CALGB 40601 trial did not collect quantitative HR staining data, and given the local determination of ERBB2/HER2 status that used ASCO/CAP guidelines but did not centrally review, it is not possible to analyze by quantitative HR or by ERBB2/HER2 determination method; this is a missed opportunity.

## Conclusions

In this cohort study of CALGB 40601, *PIK3CA* mutations were significantly associated with lower pCR rates among patients receiving trastuzumab-based therapy. *PIK3CA* mutations were also associated with worse EFS in univariable and multivariable Cox proportional hazards regression models, which appeared to be associated with HR-positive and luminal ERBB2/HER2-positive BC.

## Notes

Supplement 1. **eFigure 1.** CONSORT Diagram of DNA/RNA CALGB 40601 Population Analysis **eFigure 2.** Distribution of Different Types of *PIK3CA* Mutations **eFigure 3.** Distribution of *PIK3CA* Mutations by HR Status (A) and Intrinsic Subtype (B) in the Analytic Population **eFigure 4.** Event-Free Survival by *PIK3CA* Mutation in the Overall Population (A) and Multivariable Model Stratified by Hormone Receptor Status (B) **eFigure 5.** Event-Free Survival by *PIK3CA* Mutation in Combined Luminal A and Luminal B Breast Cancer Subgroup (A) and Multivariable Model
eMethods.

eReferences.

Supplement 2.
Data Sharing Statement
