# Supplementary material for: Association of PIK3CA Mutation With Pathologic Complete Response and Outcome by Hormone Receptor Status and Intrinsic Subtype in Early-Stage ERBB2/HER2-Positive Breast Cancer
Source: JAMA Netw Open. 2023 Dec 20;6(12):e2348814. doi: 10.1001/jamanetworkopen.2023.48814 (PMC10733807; doi:10.1001/jamanetworkopen.2023.48814)
Supplement: Supplement 1. — eFigure 1. CONSORT Diagram of DNA/RNA CALGB 40601 Population Analysis eFigure 2. Distribution of Different Types of PIK3CA Mutations eFigure 3. Distribution of PIK3CA Mutations by HR Status (A) and Intrinsic Subtype (B) in the Analytic Population eFigure 4. Event-Free Survival by PIK3CA Mutation in the Overall Population (A) and Multivariable Model Stratified by Hormone Receptor Status (B) eFigure 5. Event-Free Survival by PIK3CA Mutation in Combined Luminal A and Luminal B Breast Cancer Subgroup (A) and Multivariable Model eMethods. eReferences. [file jamanetwopen-e2348814-s001.pdf]

Supplementary Online Content

Zagami P, Fernandez-Martinez A, Rashid NU, et al. Association of *PIK3CA* mutation with pathologic complete response and outcome by hormone receptor status and intrinsic subtype in early-stage ERBB2/HER2-positive breast cancer. *JAMA Netw Open.* 2023;6(12):e2348814. doi:10.1001/jamanetworkopen.2023.48814

eFigure 1. CONSORT Diagram of DNA/RNA CALGB 40601 Population Analysis

eFigure 2. Distribution of Different Types of *PIK3CA* Mutations

eFigure 3. Distribution of *PIK3CA* Mutations by HR Status (A) and Intrinsic Subtype (B) in the Analytic Population

eFigure 4. Event-Free Survival by *PIK3CA* Mutation in the Overall Population (A) and Multivariable Model Stratified by Hormone Receptor Status (B)

eFigure 5. Event-Free Survival by *PIK3CA* Mutation in Combined Luminal A and Luminal B Breast Cancer Subgroup (A) and Multivariable Model

eMethods.

eReferences.

This supplementary material has been provided by the authors to give readers additional information about their work.

© 2023 Zagami P et al. *JAMA Network Open.*

eFigure 1. CONSORT Diagram of DNA/RNA CALGB 40601 Population Analysis

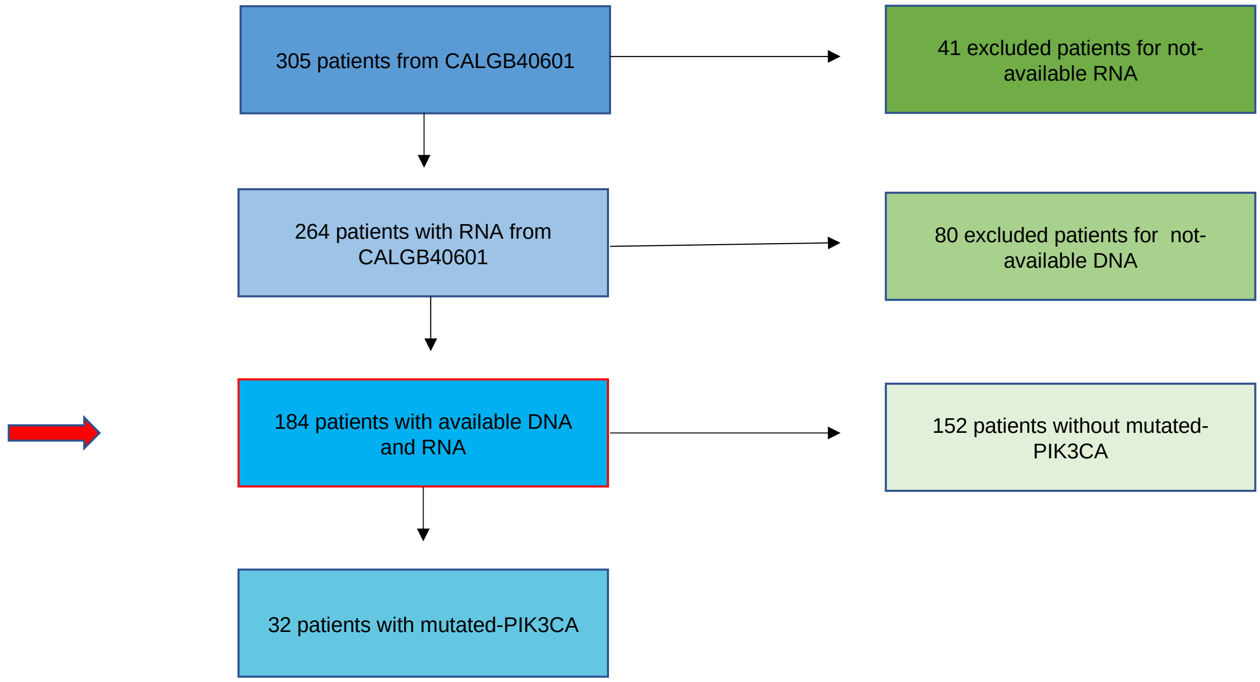

© 2023 Zagami P et al. *JAMA Network Open.*

eFigure 2. Distribution of Different Types of *PIK3CA* Mutations

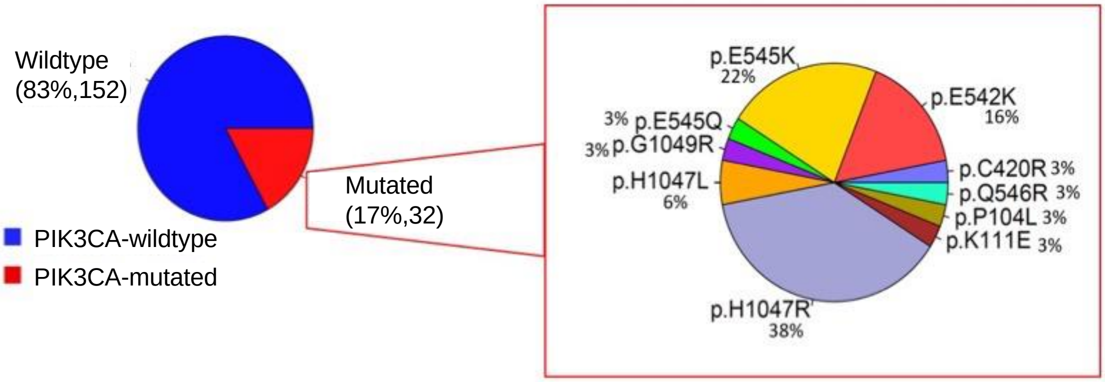

© 2023 Zagami P et al. *JAMA Network Open.*

eFigure 3. Distribution of *PIK3CA* Mutations by HR Status (A) and Intrinsic Subtype (B) in the Analytic Population

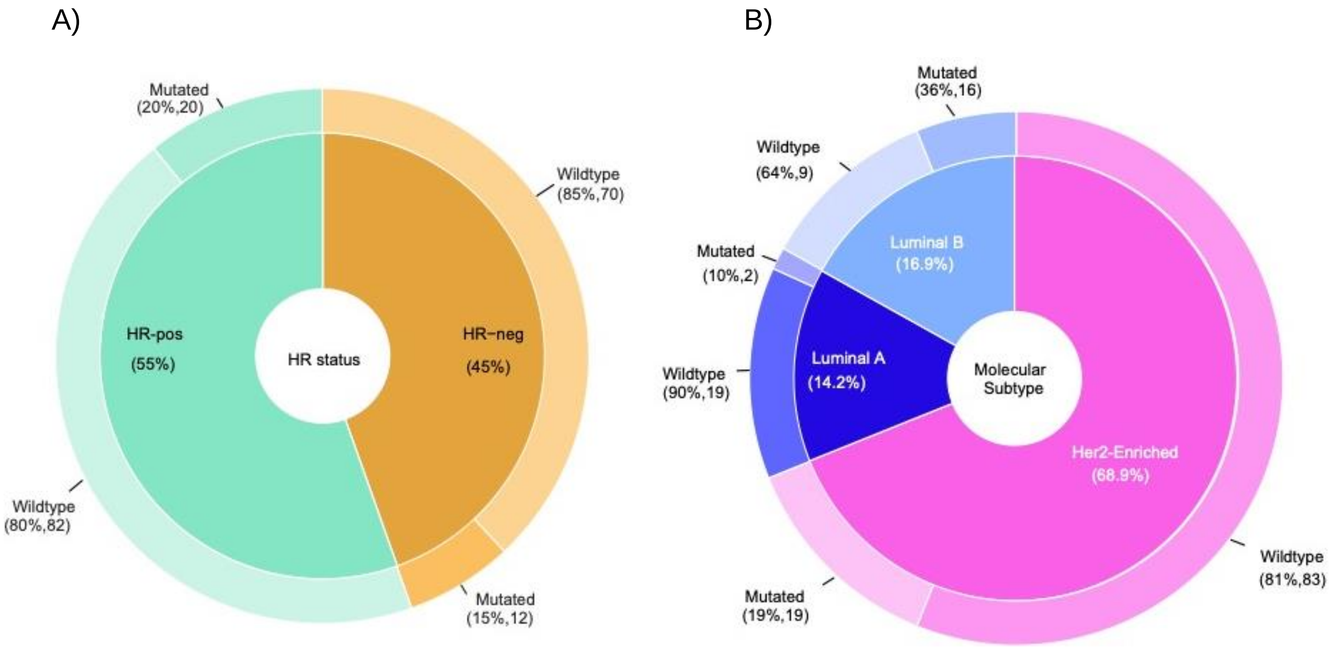

HR=hormone receptor; HR-neg=hormone receptor-negative; HR-pos=hormone receptor-positive

© 2023 Zagami P et al. *JAMA Network Open.*

eFigure 4. Event-Free Survival by *PIK3CA* Mutation in the Overall Population (A) and Multivariable Model Stratified by Hormone Receptor Status (B)

A)

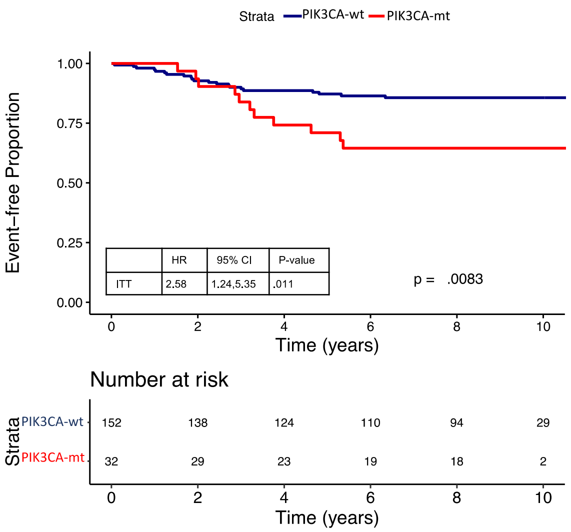

B)

	HR-positive			HR-negative		
**Characteristic**	**HR**[1]	**95% CI**[1]	**p-value**	**HR**[1]	**95% CI**[1]	**p-value**
PIK3CA						
wildtype	—	—		—	—	
mutated	4.84	1.63, 14.3	.004	1.36	0.36, 5.11	.70
Pathologic complete response						
pCR	—	—		—	—	
RD	1.79	0.60, 5.33	.297	5.05	1.36, 18.8	.016
Stage						
II	—	—		—	—	
III	2.96	0.99, 8.81	.051	1.18	0.37, 3.75	.80
Subtype						
Not HER2E	—	—		—	—	
HER2E	2.08	0.81, 5.30	.127	0.97	0.30, 3.20	>.90

[1] HR = Hazard Ratio, CI = Confidence Interval

pCR=pathological complete response; RD=residual disease wt=wildtype; mt=mutated; HER2E=HER2-enriched intrinsic subtype

© 2023 Zagami P et al. *JAMA Network Open.*

eFigure 5. Event-Free Survival by *PIK3CA* Mutation in Combined Luminal A and Luminal B Breast Cancer Subgroup (A) and Multivariable Model

A)

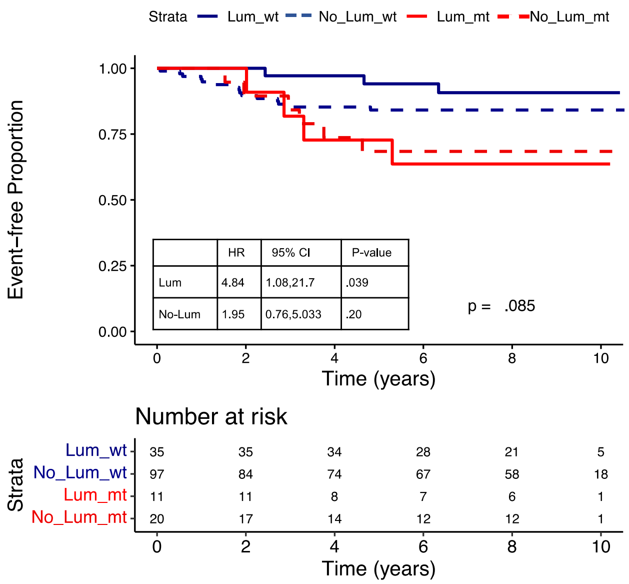

B)

	Luminal			Non-Luminal		
Characteristic	HR[1]	95% CI[1]	p-value	HR[1]	95% CI[1]	p-value
PIK3CA						
wildtype	—	—		—	—	
mutated	4.68	0.85, 25.7	.076	1.72	0.65, 4.58	.30
Pathologic complete response						
pCR	—	—		—	—	
RD	0.41	0.08, 2.26	.30	3.88	1.48, 10.1	.006
Stage						
II	—	—		—	—	
III	0.78	0.06, 9.28	.80	1.66	0.69, 4.04	.30

[1] HR = Hazard Ratio, CI = Confidence Interval

*removing normal-likes

Lum=Luminal; pCR=pathological complete response; RD= residual disease wt=wildtype; mt=mutated

© 2023 Zagami P et al. *JAMA Network Open.*

eMethods.

Tumor genomic analysis.

RNAseq libraries were made from total RNA using Illumina TruSeq® mRNA kit and sequenced on an Illumina HiSeq 2000 using a 2x50bp configuration. CALGB 40601 RNAseq FASTQ files are available on dbGAP repository (phs001570.v3.p1). Purity-filtered reads were aligned to the human reference GRCh38/hg38 genome using Spliced Transcripts Aligned to a Reference (STAR) version 2.4.2a28. Transcript (GENCODE v22) abundance estimates were generated by Salmon version 0.6.029 in '-quant' mode, based on the STAR alignments. Raw read counts for all RNAseq samples were normalized to a fixed upper quartile. RNAseq normalized gene counts were then log2 transformed, and genes were filtered for those expressed in 70% of samples. Intrinsic subtypes were obtained from RNAseq gene expression data as described before[1].

For the DNA analysis, Whole Exome Sequencing (WES) was performed at the McDonnell Genome Institute (Washington University) as previously described[2]. Briefly, using the hybrid capture exome sequencing (Nimblegen v3.0 SeqCap reagent) and matched peripheral blood mononuclear cells sequenced to average 100x depth coverage using paired-end 2x 100bp. Tumor and Normal sequences were aligned to the hg38 reference genome by BWA-mem (https://github.com/lh3/bwa) and refined using ABRA2 (https://github.com/mozack/abra2). The WES detection method was updated to the genome hg38, and identified somatic variants were called by three different methods (instead of one as previously reported[2]): Strelka2, Cadabra, and Mutect2 (https://github.com/broadinstitute/gatk/blob/master/src/main/java/org/broadinstitute/hellbender/tools/walkers/mutect/Mutect2.java). Variants were considered if they pass all of any individual caller's filters and the variant's quality score exceeded a configurable threshold (cadabra indels: 10.8, mutect2 indels: 7.0, strelka2 indels: 15.0, mutect2 snvs: 8.4, strelka2 snvs: 18.2)[3–7].

eReferences.

1. Fernandez-Martinez A, Krop IE, Hillman DW, et al. Survival, Pathologic Response, and Genomics in CALGB 40601 (Alliance), a Neoadjuvant Phase III Trial of Paclitaxel-Trastuzumab With or Without Lapatinib in HER2-Positive Breast Cancer. *Journal of Clinical Oncology*. 2020;38(35):4184-4193. doi:10.1200/JCO.20.01276

2. Tanioka M, Fan C, Parker JS, et al. Integrated Analysis of RNA and DNA from the Phase III Trial CALGB 40601 Identifies Predictors of Response to Trastuzumab-Based Neoadjuvant Chemotherapy in HER2-Positive Breast Cancer. *Clinical Cancer Research*. 2018;24(21):5292-5304. doi:10.1158/1078-0432.CCR-17-3431

3. Dobin A, Davis CA, Schlesinger F, et al. STAR: ultrafast universal RNA-seq aligner. *Bioinformatics*. 2013;29(1):15-21. doi:10.1093/bioinformatics/bts635

4. Patro R, Duggal G, Love MI, Irizarry RA, Kingsford C. Salmon provides fast and bias-aware quantification of transcript expression. *Nat Methods*. 2017;14(4):417-419. doi:10.1038/nmeth.4197

5. Bullard JH, Purdom E, Hansen KD, Dudoit S. Evaluation of statistical methods for normalization and differential expression in mRNA-Seq experiments. *BMC Bioinformatics*. 2010;11(1):94. doi:10.1186/1471-2105-11-94

6. Kim S, Scheffler K, Halpern AL, et al. Strelka2: fast and accurate calling of germline and somatic variants. *Nat Methods*. 2018;15(8):591-594. doi:10.1038/s41592-018-0051-x

7. Mose LE, Perou CM, Parker JS. Improved indel detection in DNA and RNA via realignment with ABRA2. *Bioinformatics*. 2019;35(17):2966-2973. doi:10.1093/bioinformatics/btz033

© 2023 Zagami P et al. *JAMA Network Open.*
