# Supplementary material for: Association of PIK3CA Mutation With Pathologic Complete Response and Outcome by Hormone Receptor Status and Intrinsic Subtype in Early-Stage ERBB2/HER2-Positive Breast Cancer
Source: JAMA Netw Open. 2023 Dec 20;6(12):e2348814. doi: 10.1001/jamanetworkopen.2023.48814 (PMC10733807; doi:10.1001/jamanetworkopen.2023.48814)
Supplement: Supplement 2. — Data Sharing Statement [file jamanetwopen-e2348814-s002.pdf]

Data Sharing Statement

Zagami. Association of PIK3CA Mutation With Pathologic Complete Response and Outcome by Hormone Receptor Status and Intrinsic Subtype in Early-Stage ERBB2/HER2-Positive Breast Cancer. *JAMA Netw Open*. Published December 20, 2023. doi:10.1001/jamanetworkopen.2023.48814

Data
Data available: Yes
Data types: Deidentified participant data
How to access data: dbGAP a/ o GEO
When available: With publication

Supporting Documents
Document types: None

Additional Information
Who can access the data: approved researchers per database requirements
Types of analyses: any
Mechanisms of data availability: availability will be via national databases using their mechanisms.
